# BDNF Val^66^Met and 5-HTTLPR Genotype are Each Associated with Visual Scanning Patterns of Faces in Young Children

**DOI:** 10.3389/fnbeh.2015.00175

**Published:** 2015-07-13

**Authors:** Antonios I. Christou, Yvonne Wallis, Hayley Bair, Hayley Crawford, Steven Frisson, Maurice P. Zeegers, Joseph P. McCleery

**Affiliations:** ^1^School of Psychology, University of Birmingham, Birmingham, UK; ^2^West Midlands Regional Genetics Laboratory, Birmingham Women’s Hospital, NHS Foundation Trust, Birmingham, UK; ^3^Centre for Research in Psychology, Behaviour and Achievement, Coventry University, Coventry, UK; ^4^Cerebra Centre for Neurodevelopmental Disorders, School of Psychology, University of Birmingham, Birmingham, UK; ^5^Department of Complex Genetics, NUTRIM School for Nutrition, Toxicology and Metabolism, Maastricht University, Maastricht, Netherlands; ^6^Center for Autism Research, Children’s Hospital of Philadelphia, Philadelphia, PA, USA

**Keywords:** BDNF Val^66^Met, 5-HTTLPR, eye movement, emotional face, facial features, affective neuroscience, early childhood

## Abstract

Previous studies have documented both neuroplasticity-related BDNF Val^66^Met and emotion regulation-related 5-HTTLPR polymorphisms as genetic variants that contribute to the processing of emotions from faces. More specifically, research has shown the BDNF Met allele and the 5-HTTLPR Short allele to be associated with mechanisms of negative affectivity that relate to susceptibility for psychopathology. We examined visual scanning pathways in response to angry, happy, and neutral faces in relation to BDNF Val^66^Met and 5-HTTLPR genotyping in 49 children aged 4–7 years. Analyses revealed that variations in the visual processing of facial expressions of anger interacted with BDNF Val^66^Met genotype, such that children who carried at least one low neuroplasticity Met allele exhibited a vigilance–avoidance pattern of visual scanning compared to homozygotes for the high neuroplasticity Val allele. In a separate investigation of eye gaze towards the eye versus mouth regions of neutral faces, we observed that short allele 5-HTTLPR carriers exhibited reduced looking at the eye region compared with those with the higher serotonin uptake Long allele. Together, these findings suggest that genetic mechanisms early in life may influence the establishment of patterns of visual scanning of environmental stressors, which in conjunction with other factors such as negative life events, may lead to psychological difficulties and disorders in the later adolescent and adult years.

## Introduction

Rapid responses to threatening stimuli have been proposed to have a strong evolutionary component, with threat stimuli requiring urgent facilitation of cognitive and behavioral responding (Öhman and Mineka, 2001; Holmes et al., 2009; Becker et al., 2011). Due to the importance of the human face as an explicit signal for possible aggression and, therefore, in the detection of risk for immediate social threat, the use of facial expressions of anger has come to be used as a reliable index of early fear-related social affectivity in this area of research. For example, an “anger superiority effect” has emerged to reflect a documented pattern of preferential processing of angry faces compared with facial expressions of other emotions (Hansen and Hansen, 1988; Holmes et al., 2009; Öhman et al., 2010). The majority of the studies that have examined visual scanning of angry versus other emotional faces have supported the existence of this superiority effect, reporting quicker speed of detection for angry faces compared with both happy and neutral facial expressions (Gilboa-Schechtman et al., 1999; Fox and Damjanovic, 2006; Horstmann and Bauland, 2006; Lipp et al., 2009; Pinkham et al., 2010; Öhman et al., 2010; Susa et al., 2012).

Recent accounts have suggested a dual-stage processing of emotional stimuli, with anxious individuals exhibiting increased attentional orientation toward negatively valenced stimuli during early stages of processing (e.g., 0–500 ms) compared with control participants, while simultaneously exhibiting an avoidant looking pattern for the same negative stimuli at a later stage of processing (e.g., 1000–1500; Koster et al., 2005). This pattern of results, known in the field as vigilance–avoidance, is believed to reflect automatic attentional orienting to threat-related information followed by strategic visual avoidance in an effort to suppress negative arousal resulting from continued exposure to the negative stimuli (Mogg et al., 2004).

In addition to investigations of visual scanning behavior toward negative emotional expressions in faces, a considerable body of research has focused on investigating eye gaze patterns toward specific facial features that have been suggested to be critical for successful social interaction. Most notably, attending to the eyes has been identified as critical for successful facial identification (Gold et al., 2008), as well as for the effective detection and classification of another individual’s facial emotions and intentions [Baron-Cohen et al., 1997; but see also Dailey and Cottrell (1999) and Blais et al. (2012)]. In addition, typically developing children have been found to focus quickly on the eye region of the human face from very early in life (Nation and Penny, 2008; Shepherd, 2010). Furthermore, healthy individuals have been observed to first fixate on the eyes, and to subsequently spend relatively more time looking at the eye region compared with the mouth region of the face [for a review, see Itier and Batty (2009)]. Affected young populations, such as children with autism, have been found to exhibit an atypical pattern of processing these facial features, spending more time looking at the mouth than the eyes during face scanning in some studies (Klin et al., 2002; Pelphrey et al., 2002; de Wit et al., 2008; Norbury et al., 2009; Kliemann et al., 2010). However, a recent review of eye-tracking studies examining social-specific visual attention in ASD has confirmed the evidence that suggest atypicalities on the visual scanning of the most significant social cues but also reported that this behavioral pattern is not generalized across various contexts, such as in the case of enhanced child-directed speech (Guillon et al., 2014). Emerging research has also highlighted atypical looking to the eye region of faces in individuals with Fragile X syndrome, a genetically defined neurodevelopmental disorder associated with social and communication impairments, as well as social anxiety and reduced amygdala volume [Crawford et al., 2014; see also Dalton et al. (2008), Holsen et al. (2008), Farzin et al. (2009), and Hazlett et al. (2012)]. Together, these findings suggest that reduced eye looking during static face scanning may be closely associated with individual differences in social anxiety and associated neural systems (Crawford et al., 2014).

Although recent evidence provides insight into the time-course of face scanning behavior of anxious adults [e.g., Rohner (2002), Calvo and Avero (2005), and Garner et al. (2006)], the ways in which neurobiological mechanisms may contribute to the early manifestation of these behaviors are currently poorly understood. According to the Differential Susceptibility Hypothesis, individuals are differentially affected by their experiences over the course of development, which is determined by pre-existing elevated biological sensitivity factors such as genetic vulnerabilities that are determined from normal variations in candidate genes (Belsky, 1997; Belsky et al., 2007).

For example, there has been increasing scientific consensus in recent years to support the involvement of BDNF Val^66^Met variants in modulating behavior, including stress reactivity and depressive symptomatology. Brain-derived neurotrophic factor (BDNF) is a secreted protein present in the human brain that is part of the neurotrophin growth factor family and has been observed to be involved in the regulation of survival and differentiation of neurons, as well as synaptic plasticity (Lu, 2003). Most notably, the BDNF single nucleotide polymorphism (SNP) Val^66^Met results in a change from Guanine (G) to Adenine (A) at nucleotide position 196 in the protein coding sequence of the gene, as well as subsequent change in amino acid from valine to methionine at position 66 (rs6265). This ultimately leads to decreased availability of BDNF in the brain due to decreased secretion of the variant form of BDNF (Egan et al., 2003). Relevant to emotion regulation, neuroimaging studies have found healthy adults carrying the Met allele to exhibit functional neural over-activation in response to emotional stimuli (Montag et al., 2008; Lau et al., 2010). The results of studies of children also suggest that the BDNF Met allele may act as a susceptibility factor for affective disorders [e.g., Beevers et al. (2009)], especially in combination with early life stressors (Gatt et al., 2009). Despite the consistency in the findings discussed here, however, there remains significant controversy in the literature regarding BDNF Val^66^Met [for a recent review, see Groves (2007)].

In addition to the putative role of BDNF and associated neuroplasticity in affective responses to emotional faces, associations between common genetic variation in serotonin transporter genes and individual differences in visual scanning of emotional faces have also been observed [e.g., Battaglia et al. (2005) and Lau et al. (2009)]. In particular, a common polymorphism in the 5-HT promoter region, 5-HTTLPR, involved in the transport of serotonin to the presynaptic neuron, has been identified as a reliable indicator of psychological maladjustment. This polymorphism is represented by two variants, a short (S) allele and a long (L) allele, with the short allele associated with significant decreases in serotonin reuptake (Lesch et al., 1996). These alleles produce three genotypes (short/short, short/long, long/long). In combination with exposure to life-threatening situations, individuals carrying at least one copy of the Short allele have been reported to be at increased susceptibility for negative cognitive, behavioral, and neurophysiological outcomes [Caspi et al., 2003; Mercer et al., 2012; Xie et al., 2012; Disner et al., 2013; see also Boll and Gamer (2014) and Christou et al. (2015)]. However, discrepancies still exist in the field regarding the particular role of variations in the serotonin gene in moderating responses to environmental events [for a meta-analysis, see also Karg et al. (2011) and Risch et al. (2009)]. From a developmental perspective, children as young as 9 years of age carrying the 5-HTTLPR Short allele have been found to exhibit greater neural activation in response to fearful and angry faces than children homozygous for the Long allele, in various brain regions previously linked to attentional control in adults (Thomason et al., 2010). In line with this neurophysiological evidence, a range of behavioral studies in both children and adults have measured behavioral reaction times, and reported that the presence of two copies of the high activity Long 5-HTTLPR allele is associated with positive affectivity (shorter reaction times) toward happy facial stimuli compared to neutral facial stimuli [for a review, see Homberg and Lesch (2010)], suggesting the existence of a protective factor against affective psychopathology in homozygotes for the Long allele.

Three hypotheses were tested as part of this study. Taking into account previous evidence suggesting a moderating role of the BDNF Val^66^Met for reactivity (Montag et al., 2008; Schofield et al., 2009; Lau et al., 2010), it was hypothesized that the low neuroplasticity Met BDNF allele would be significantly associated with vigilance–avoidance patterns in the time spent looking toward the facial expressions of anger, compared to the high neuroplasticity Val/Val genotype. Furthermore, considering evidence suggesting modulation of reactivity in response to facial emotions by 5-HTTLPR genotype (Thomason et al., 2010), we hypothesized that carriers of at least one low serotonin uptake 5-HTTLPR Short allele would similarly display a vigilance–avoidance pattern in response to angry facial expression. A third hypothesis was that carriers of at least one 5-HTTLPR Short allele would exhibit an avoidance pattern in overall time spent looking at the eyes versus the mouth region of neutral face pairs, compared with the high serotonin uptake Long/Long genotype. Finally, we tested the hypothesis that carriers of at least one BDNF Met allele would similarly exhibit an avoidance pattern in overall time spent looking at the eyes versus the mouth of neutral face pairs, compared with individuals homozygous for the Val allele.

## Materials and Methods

### Participants

Forty-nine children from Caucasian ancestry participated in the study (24 males; 25 females; *M* age in months = 60.26, SD = 11.80, age range: 4–7 years of age). Participants were recruited through a local community research participation advertisement/outreach program at the local University from a pool of 70 children that have originally genotyped for the needs of previous neuroimaging genetics study on our lab (Christou et al., 2015). Parents or guardians of all participants reported that the child had no history of a neurological or psychiatric disorder, with normal or corrected to normal vision. All participants scored above 75 on the British Ability Scales (BAS-II; Elliot et al., 1996), a standardized assessment of intelligence/developmental age and abilities, equivalent to IQ scores. Informed written consent was obtained from the parents/guardians of all participants prior to participation in the study. In addition, children aged 7 provided written assent to participate in the study. Families were provided with compensation of £10 toward their travel expenses. Ethical consent was gained from the local University Ethical Committee.

### Emotion regulation measures

For the assessment of children’s emotional regulation abilities, the Child Behavioural Checklist was used (CBCL; Achenbach and Rescorla, 2001). The CBCL includes two different versions: the Early Years version (for children between 1 ½ and 5 years of age) and the School Age version (for children and adolescents aged 6–18 years). Both the Early Years and School Age versions were used here. Both versions of CBCL have two main subscales which are structurally independent from each other (Achenbach and Rescorla, 2001), which map externalizing (e.g., aggression, impulsivity, and hyperactivity) and internalizing symptoms (e.g., distressful and over controlled behaviors). Higher total scores in each subscale or in each symptom category suggest the existence of more problematic behaviors, with *t*-scores of more than 60 to be considered in the range of clinical significance. Therefore, participants who scored higher than the cut-off point were excluded from further analyses.

### Eye-tracking assessment

#### Stimuli

A total of 80 trials of colored happy-neutral, angry-neutral, and neutral–neutral face pairs constructed the experiment. All the face stimuli were selected from the MacBrain Face Stimulus Set^1^ (Tottenham et al., 2009) and were matched in terms of gender, race, and age. Available validity data for the MacBrain Face Stimulus Set in both children and adults have been reported high inter-rater agreement for the emotion that is displayed in these facial expressions (Tottenham et al., 2009). Pairs of faces were presented simultaneously side-by-side, with emotional faces presented equally on the right and the left side of the screen. In order to determine whether increased or reduced fixation duration toward the emotional faces (critical trials) resulted from heightened orientation, difficulty in disengaging from emotional stimuli, or both, the experiment was constructed using baseline neutral–neutral face pair trials (baseline; *N* = 70) and critical trials of emotional-neutral face trails (i.e., five angry-neutral pairs; five happy-neutral pairs; *N* = 10). The experiment started with seven baseline trials (pairs of neutral–neutral faces), where at least four baseline trials were presented between the critical trials (pairs of emotional-neutral faces). Baseline and critical trials were pseudorandomly allocated across the experiment in line with previous behavioral studies [e.g., Mogg et al. (2004), Salemink et al. (2007), Arndt and Fujiwara (2012), and Crawford et al. (2014)]. The eye-tracking experiment was programed using Experiment Builder software for EyeLink (SR Research, ON, Canada). The facial stimuli consisted of 38 color photographs of male and female faces^1^ (1024 × 768 pixels) depicting one of three expressions (neutral, happy, and angry). Although some of the neutral face pairs were repeated across the experiment, the neutral face stimuli used during the critical trials were not used elsewhere during the experiment. Therefore, face familiarity did not affect face preferences during critical trials.

**Figure 1:**
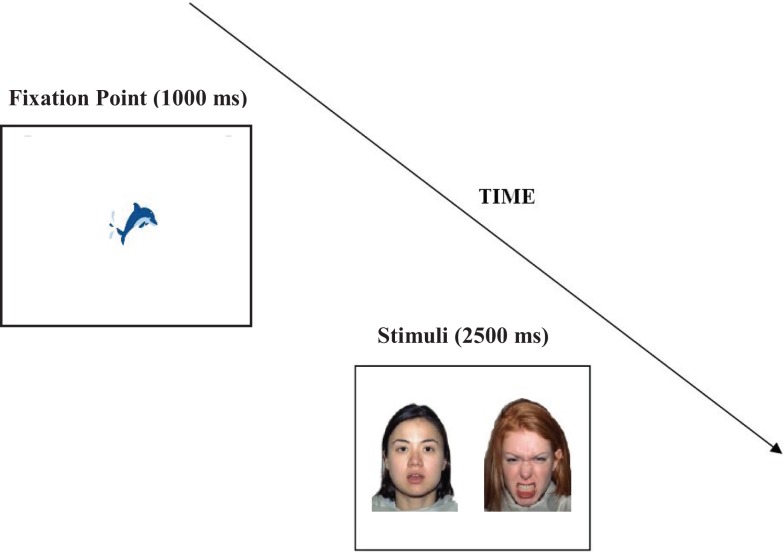
**An example of the face stimuli pairs used in the eye-tracking experiment and an illustration of a trial structure**.

Each trial began with a fixation point (in the shape of an animated dolphin), measuring 2.81 × 2.08 ° of visual angle in the middle of the screen which was displayed for 1000 ms (except in the case of mini calibration; see Section “Procedure”). This was followed by a pair of faces presented side by side against a white background for 2500 ms. The inter trial interval was 1000 ms (see Figure 1). The gap between the two faces was 7.2° of visual angle. Each stimulus pair was presented with a visual angle of 14.3 × 18.6°.

#### Procedure

Participants’ eye movements were recorded using an Eyelink 1000 Tower Mount eye-tracking system and the stimuli were presented on a 19-inch CRT with a resolution of 1024 × 768 pixels. The eye-tracker sampled eye position at 500 Hz (i.e., every 2 ms). Average spatial accuracy is between 0.25° and 0.5° of visual angle. Participants were seated in a dimly lit room, 60 cm away from the display screen, and they had their head positioned against a head rest and their chin placed on a chinrest to minimize the possibility of movements. Viewing was binocular, but only data from the right eye were collected. During calibration, the EyeLink recorded the eye position at five target locations, providing the required reference data for computing gaze positions to ensure a point of fixation error rate of <0.5°. A mini calibration was repeated every five trials in order to ensure that eye movement data were adjusted for small-scale movement of the head. In the case of unsatisfactory eye-tracking, a 5-point calibration was repeated.

### Data collection procedures

Children were told that they were going to see a range of interesting photos on a computer screen, while a special camera recorded their eye movements. The eye-tracking and behavioral assessments took place in one laboratory visit.

#### Analysis of Behavioral Data

All children in this study had *t*-scores of <60 (below subclinical range) at the CBCL. Therefore, no participants were excluded due to elevated clinical symptomatology. Raw scores from the two clusters of symptomatology (i.e., internalizing and externalizing symptoms) were used for statistical analysis from both CBCL versions following the authors’ guidelines (Achenbach and Rescorla, 2001, p. 89) and as previously reported in studies with children [e.g., Stanger et al. (2011)]. Higher total scores in each CBCL subscale suggest the existence of more problematic behaviors. For the measures of cognitive abilities (BAS II), mean standardized IQ scores were calculated.

#### Reduction of Eye-Tracking Data

Fixations were calculated via online detection analysis when eye movement met the following four criteria: (a) velocity threshold of 30°/s, b) a motion threshold of.1°, (c) a 8000°/s^2^ acceleration threshold, (d) and the pupil was not missing consecutively for three or more times from a sample. Trials were classified as “invalid” if a child did not look at all at the faces during the trial. In addition, if more than 40% invalid trials were evident, the participant’s data were excluded from further analyses. No participant met this exclusion criterion; therefore, all 49 participants provided valid eye-tracking data.

For analyses, each 2500 ms trial was divided into five 500 ms intervals. The relative mean proportions of viewing time for the angry and happy faces were then calculated for each 500 ms time interval of watching during the critical trials. This was done by subtracting the overall dwell time of the neutral stimuli (for each critical trial) from the overall dwell time looking at the emotional (happy or angry) face. This was done separately for each subject and for each happy and angry critical trial. Average dwell time of looking for each emotion type (i.e., angry, happy) was later calculated for each subject. Two additional regions of interest (RoIs) for the eyes and mouth region were identified. For this analysis, the neutral only/baseline pairs were used, where the coordinates of gaze for each eye as well as the mouth region were identified and extracted using the EyeLink Data viewer software. The overall amount of time spent (in ms) looking at the eye and mouth regions was divided by the amount of time spent (in ms) looking at the whole neutral face. This was done separately for each neutral baseline trial (for the overall 2500 ms), and then averaged across the baseline trials for each participant.

For both of these analyses, after the subtraction, positive values represented a visual preference for the emotionally expressive face (versus neutral) or facial feature, and negative values represented visual patterns that relate to avoidance behavior for the emotionally expressive face (versus neutral) or facial feature.

At this stage, it is worth underlining that eye-tracking studies have examined additional patterns of eye movements over time, such as mean proportion of fixations [e.g., Garner et al. (2006) and Gamble and Rapee (2010)], where vigilance–avoidance patterns of attention to angry faces have been reported for individuals with social phobia. However, the absence of continuous eye gaze data recorded from overall dwell time in these studies has resulted in significant criticism [e.g., Schofield et al. (2012)], suggesting that longer periods of looking time, as opposed to shorter (e.g., 500 ms; Schofield et al., 2012), provide the most reliable evidence for vigilance–avoidance patterns of visual scanning. To this end, the present study, in line with the procedures of other eye tracking studies with young populations [e.g., de Wit et al. (2008), Farzin et al. (2009), and Crawford et al. (2014)], reports the overall time spent looking at emotional faces as well as on the additional RoIs identified.

### Analysis of genetic material

Genomic DNA was extracted from saliva samples using the Oragene OG-500 self-collection kit (Oragene, DNA Genotek Inc., Canada), according to the manufacturer’s recommendations. DNA concentrations ranged from 65 to 962 ng/ul and the 260/280 ratio was between 1.8 and 2 for all samples. Genotyping results were successfully obtained for all 49 subjects.

#### BDNF Val^66^Met Genotyping

Direct bidirectional sequencing was used to genotype the SNP within the BDNF gene (rs6265). PCR primers were designed to flank the polymorphism producing a 249 bp amplification product. Sequences of the primers are as follows: forward AAACATCCGAGGACAAGGTG and reverse AGAAGAGGAGGCTCCAAAGG. PCR was performed using Megamix PCR solution (supplied by Microzone UK Ltd.) in a total volume of 25 ul containing 25 pmol of each primer. An initial denaturation step at 95°C for 5 min was followed by 30 cycles of PCR (95°C 1 min, 58°C 1 min, 72°C 1 min) and then a final extension at 72°C for 10 min. PCR products were purified using Exonuclease I and Shrimp Alkaline Phosphatase (according to manufacturer’s instructions). Ten microliters sequencing reactions were generated, containing 0.25 ul BigDye Terminator (v3.1, Applied Biosystems), 1.9 ul molecular grade water, 3 pmol of forward or reverse primer, and 1 ul purified 5-HTTLPR PCR amplicon (diluted 1 in 2). Cycle conditions for sequencing included an initial denaturation step at 95°C for 5 min followed by 30 cycles of PCR (95°C 30 s, 50°C 10 s, 60°C 4 min) and reaction products were purified using CleanSEQ^®^ beads (Agencourt) in a 1:1 ratio as described by the manufacturer. Products were resuspended in 70 ul molecular grade water and analyzed on a 3730 Genetic Analyzer (Applied Biosystems).

Allele frequencies for the BDNF Val^66^Met was *n* = 24 (25.5%) for Mel alleles and *n* = 74 (75.5%) for Met alleles, respectively. To this end, three genotype groups were generated, one with Met allele homozygotes (i.e., M/M; *N* = 3), heterozygotes V/M (*N* = 18), as well as homozygotes for the Val allele (i.e., V/V; *N* = 28). However, taken the small sample of participants homozygous for the low activity Met allele (*N* = 3) and the previous evidence associating the presence of at least one Met activity with behavioral outcomes [e.g., Wichers et al. (2008)], here, carriers of at least one Met allele [i.e., heterozygotes (Met/Val) and homozygotes for the Met allele (Met/Met)] were grouped in one “Met allele carriers” group (i.e., M/−; *N* = 21).

#### 5-HTTLPR Genotyping

Direct bidirectional sequencing was used to genotype the 5-HTTLPR polymorphism. The region containing the 43 bp insertion polymorphism was amplified using primers described in Hu et al. (2006) producing a 528 bp amplification product from the L allele and a 485 bp product from the S allele. PCR was performed using Megamix PCR solution (supplied by Microzone UK Ltd.) in a total volume of 25 ul, containing 25 pmol of each primer and 3 ul of betaine. An initial denaturation step at 95°C for 5 min was followed by 30 cycles of PCR (95°C 1 min, 58°C 1 min, 72°C 1 min) and then a final extension at 72°C for 10 min. PCR products and the remaining sequencing procedure were the same as described for the BDNF Val^66^Met genotyping.

Allele frequencies across participants for 5-HTTLPR were *n* = 59 (42.1%) for Short allele and *n* = 81 (57.9%) for Long Allele. We classify three groups of participants: one with homozygous for the Short allele (S/S; *N* = 10), one with heterozygotes (S/L; *N* = 22), and one with participants homozygous for the Long allele (L/L; *N* = 17). Similar to the BDNF Val^66^Met genotype, carriers of at least one Short allele [i.e., Heterozygotes (S/L), and homozygotes for the Short allele (S/S) were grouped in one “Short allele carriers” group (i.e., S/−; *N* = 32) and compared with the remaining homozygous participants for the high serotonin uptake Long allele (L/L; *N* = 17). Hardy–Weinberg Equilibrium (HWE) for both 5-HTTLPR and BDNF Val^66^Met genotype was calculated using a tool available online (http://www.tufts.edu/~mcourt01/Documents/Court%20lab%20-%20HW%20calculator.xls).

### Statistical analyses

#### Preliminary Analyses

Descriptive statistics were conducted in order to describe the sample’s demographic characteristics such as, gender, age, and distribution of cognitive abilities. Raw data from the behavioral and cognitive scales were examined for normality using Kolmogorov–Smirnov tests. The CBCL subscales did not meet criteria for normal distributions (Kolmogorov–Smirnov, *p* < 0.05). Therefore, to further examine possible correlations between age, gender, IQ, and scores on the behavioral measures, Spearman’s Rho non-parametric correlations coefficients tests were also performed. Moreover, Pearson correlation analyses were conducted to determine if a correlation among demographic characteristics or cognitive performance and genotype group was evident, and Spearman correlation analyses were conducted to investigate possible correlations between BDNF Val^66^Met and 5-HTTLPR Genotypes and demographic, cognitive, and affective symptomatology in the sample.

#### Behavioral Ratings and Eye Gaze Patterns

The primary behavioral analysis examined whether children’s behavioral scores were correlated with fixation duration toward particular emotional faces and fixation duration toward facial features. For the cognitive abilities (BAS II) measures, mean standardized IQ-scores were assessed. Furthermore, correlation analyses were conducted to investigate possible correlation between dwell time looking at the emotional faces and participants’ demographic characteristics for each emotion and face feature separately, which showed no effect of age, developmental age, or gender in predicting overall looking time.

#### Genetics and Visual Scanning

To assess looking preferences toward and away from the emotional faces, overall dwell time spent fixating on the emotional face minus the overall dwell time spent fixating on the accompanying neutral face was computed for five time intervals: 0–500 ms (*T*_1_), 501–1000 ms (*T*_2_), 1001–1500 ms (*T*_3_), 1501–2000 ms (*T*_4_), and 2001–2500 ms (*T*_5_). A 2 (Emotion: positive versus negative) × 5 (Time: 0–500 versus 501–1000 versus 1001–1500 versus 1501–2000 versus 2001–2500 ms) mixed analysis of variance (ANOVA) with gender (female, male) and genotype (BDNF V/V versus M/−; 5-HTTLPR L/L versus S/−) as between-groups variables was conducted. All within subjects effects that violated the assumption of sphericity were adjusted using the Greenhouse–Geisser correction. To further evaluate the time course of attention, independent samples *t*-tests were conducted to determine whether there was a looking preference toward or away from the emotional images of a specific genotype group at any of the 500 ms time intervals. This was done for each SNP (BDNF Val^66^Met and 5-HTTLPR) and each facial expression (happy and angry), separately, after the initial ANOVA. When the data did not satisfy Kolmogorov–Smirnov tests for normality, Mann–Whitney *U* tests were performed instead of *t*-tests.

To investigate looking preferences toward the eye and mouth regions, a separate two-way mixed ANOVA with the repeated factor RoI (eyes, mouth) and genotype group (i.e., S/− versus L/L; M/− versus V/V) and gender as independent factor was conducted to examine gaze behavior for each face region for the baseline trials only (neutral–neutral face pairs). After the omnibus ANOVA, and because eye gaze data were non-normally distributed, a Mann–Whitney *U* test was conducted to investigate the 5-HTTLPR genotype effects on overall viewing time for the eye and mouth regions, respectively. As a secondary analysis, the original ANOVA was repeated using with additional RoIs as repeated factor (eyes, mouth, forehead, nose, cheeks, chin) and genotype group (i.e., S/− versus L/L; M/− versus V/V) and gender as independent factor (see also Figure S1 in Supplementary Material for an example of RoIs selection).

## Results

### Demographic characteristics

Tables 1 and 2 present participants’ main demographic characteristics, including gender, age, and cognitive abilities. Correlation analyses did not reveal any significant correlation between demographic characteristics and behavioral measures, or correlations between demographics, early symptomatology, and genotype. Moreover, *t*-tests showed that the two 5-HTTLPR genotype groups did not differ in terms of age [*t*(47) = −0.264, *p* = 0.793], gender [*t*(47) = 0.994, *p* = 0.325], IQ [*t*(47) = −1.17, *p* = 0.245], developmental age [*t*(47) = −0.245, *p* = 0.808], or other behavioral measures. Similarly, the two BDNF genotype groups did not differ in terms of age [*t*(47) = 0.107, *p* = 0.915], gender [*t*(47) = 0.162, *p* = 0.872], IQ [*t*(47) = −0.427, *p* = 0.671], or developmental age [*t*(47) = −0.223, *p* = 0.824]. BDNF Val^66^Met [*x*^2^(1) = 0.002, *p* = 0.962] and 5-HTTLPR genotype frequencies [*x*^2^(1) = 0.340, *p* = 0.559] were in Hardy–Weinberg equilibrium.

**Table 1:** **Sample size and demographic characteristics of sample**.

*N*		49
Gender	% Male (*N*)	48.9 (24)
	% Female (*N*)	51.1 (25)
Handedness	% Right (*N*)	77.3 (39)
	% Left (*N*)	22.7 (10)
Chronological age (months)	Mean (SD)	60.26 (11.80)
	Range	43–80

**Table 2:** **Participants general and age-equivalent cognitive abilities**.

**Chronological age (months)**	Mean (SD)	60.26 (11.80)
	Range	30
Overall ability	Mean (SD)	106.67 (8.95)
	Range	39
Verbal ability	Mean (SD)	102.86 (13.83)
	Range	64
Non-verbal ability	Mean (SD)	110.73 (14.01)
	Range	54
**Developmental age (months)**	Mean (SD)	63.99 (13.16)
	Range	45
Developmental verbal ability (months)	Mean (SD)	64.92 (15.59)
	Range	60
Developmental non-verbal ability (months)	Mean (SD)	66.67 (15.55)
	Range	61

### Behavioral effects in fixation duration

Pearson correlation analyses revealed a negative correlation between externalizing symptomatology and children’s age (*r* = −0.362, *p* = 0.011). No further relationships of participants’ demographic characteristics in cognitive development or early affective symptoms were observed. Furthermore, Spearman’s Rho correlations showed a significant positive correlation between internalizing and externalizing symptoms (*r* = 0.524, *p* < 0.001). In addition, since the eye movement data varied in terms of normality across different time points of processing (i.e., Happy *T*_2_, *T*_3_, *T*_4_ and Angry *T*_2_, *T*_5_ were *p* > 0.005; Happy *T*_1_, *T*_5_ and Angry *T*_1_, *T*_3_, *T*_4_ were *p* < 0.05 in Kolmogorov–Smirnov test of normality), both parametric and non-parametric correlation analyses were conducted with CBCL subscales and looking dwell time spent for each time point and each type of emotion separately. These analyses revealed no significant correlations.

### Genotype effects in fixation duration for emotional expressions

A 2 (Emotion: positive versus negative) × 5 (Time: 0–500 versus 501–1000 versus 1001–1500 versus 1501–2000 versus 2001–2500 ms) mixed ANOVA with Gender (female, male) and Genotype (BDNF M/− versus V/V) as between-groups factors revealed a main effect of Emotion, [*F*(1, 45) = 7.10, $\eta_{\text{p}}^{2}=0.13$, *p* = 0.011], a main effect of Time [*F*(4, 180) = 46.89, $\eta_{\text{p}}^{2}=0.75$, *p* < 0.001], and a two-way Emotion by Time interaction [*F*(4, 180) = 13.07, $\eta_{\text{p}}^{2}=0.53$, *p* < 0.001]. In terms of genotype effects, a two-way Time by BDNF Genotype [*F*(4, 180) = 4.01, $\eta_{\text{p}}^{2}=0.08$, *p* = 0.004], as well as a three-way Emotion by Time by BDNF genotype interaction [*F*(4, 45) = 3.52, $\eta_{\text{p}}^{2}=0.07$, *p* = 0.009] were evident. No further interaction effects were observed.

To further delineate the observed Time by BDNF genotype effect, the dwell time at each of the five time points was averaged across the two emotions. A Kolmogorov–Smirnov test of normality showed that the averaged data at each time point were normally distributed (*p* > 0.005); therefore, *t*-tests were conducted at each time point of visual scanning averaged across the two emotions. This analysis revealed a significant difference between the two genotype groups (i.e., M/− versus V/V) on the time spent looking at emotional stimuli during *T*4 [*t*(47) = −0.205, *p* < 0.05)]. Moreover to delineate the three-way Emotion by Time by BDNF interaction, follow up analyses were conducted to determine whether there was a preference toward or away each emotion at each of the time intervals. Because a Kolmogorov–Smirnov test revealed that the relative viewing time between stimuli in specific time points were normally distributed (e.g., Happy *T*_2_, *T*_3_, *T*_4_ and Angry *T*_2_, *T*_5_ where *p* > 0.005; where Happy *T*_1_, *T*_5_ and Angry *T*_1_, *T*_3_, *T*_4_ where *p* < 0.05 in Kolmogorov–Smirnov test of normality), this analysis was followed up with complementary parametric and non-parametric analyses at each Time Point separately.

**Figure 2:**
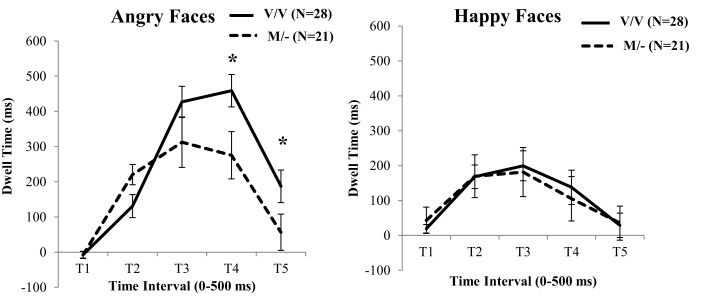
**BDNF genotype differences for fixation durations to facial expressions of Anger versus Neutral (left) and Happiness versus Neutral (right)**. Carriers of at least one Met allele fixated angry face more at the early stages, but later exhibited an avoidance pattern of attention to these faces. Error bars denotes 1 SEM. *p < 0.05.

For the time points with not-normally distributed data, a Mann–Whitney *U*-test revealed a significant difference between the two BDNF genotypes in the dwell time toward the facial expressions of Anger at *T*_4_ (*U* = 157.00, *p* = 0.010; see Figure 2; Table S3 in Supplementary Material). Moreover for the normally distributed time points, a *t*-test for *T*_5_ was shown that the carriers of the low neuroplasticity Met allele spent significantly less time looking at the angry faces [*t*(47) = −2.10, *p* = 0.041], which was absent for the happy faces. By contrast, carriers of two copies of the Val allele exhibited an increase in time looking to the angry faces.

**Figure 3:**
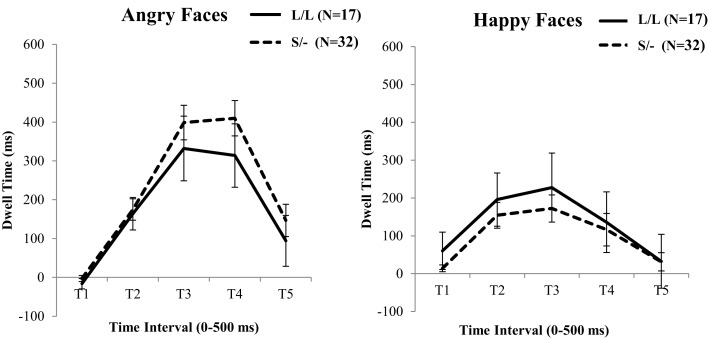
**5-HTTLPR genotype differences in fixation duration to facial expressions of Anger versus Neutral (left) and Happiness versus Neutral (right)**. Genotype groups do not differ at any time point across the two types of emotional faces. Error bars denotes 1 SEM.

In continuing, the above ANOVA analysis was repeated with the 5-HTTLPR genotype (i.e., L/L versus S/−) as a between factor. Contrary to the BDNF genotype effects, this analysis did not show a significant Time by 5-HTTLPR [*F*(1, 45) = 1.35, $\eta_{\text{p}}^{2}=0.00$, *p* = 0.857], or an Emotion by Time by 5-HTTLPR interaction [*F*(1, 45) = 0.33, $\eta_{\text{p}}^{2}=0.13$, *p* = 0.011; see Figure 3]. No further effects were detected from this analysis.

### Genotype effects on gaze patterns

**Figure 4:**
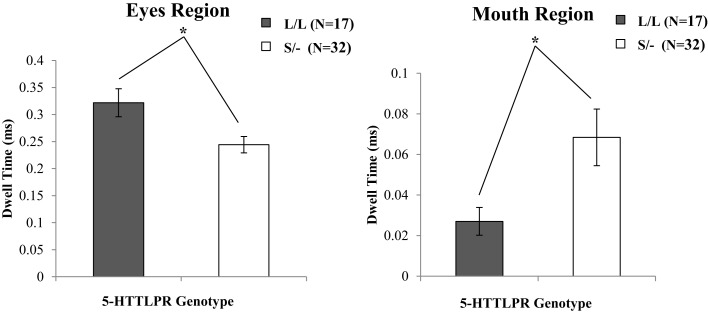
**The proportion of time spent among 5-HTTLPR genotype groups looking at the eye and mouth regions relative to the rest of the face during baseline trials**. Carriers of at least one Short allele fixated the eye region less, but spent more time looking the mouth region of neutral faces. Error bars denote 1 SEM. *p < 0.01.

A two-way mixed ANOVA, with the repeated factor RoI (eyes, mouth) and genotype group (V/V, M/−; L/L, S/−) and gender as independent factors, examined gaze behavior for each face region on the baseline trials. There was a significant effect of RoI for the face areas of interest [*F*(1,45) = 126.11, *p* < 0.001], whereby children spent more time looking at the eyes region of the neutral faces (see also Table S4 in Supplementary Material). However, a significant interaction between RoI and BDNF genotype group was not evident [*F*(1,45) = 0.74, $\eta_{\text{p}}^{2}=0.01$, *p* = 0.393]. By contrast, the 5-HTTLPR genotype shown a significant interaction between RoI and genotype group [*F*(1,45) = 7.25, $\eta_{\text{p}}^{2}=0.13$, *p* = 0.010]. To further examine the interaction effects observed in the ANOVA, and given that the data for the eyes region met normal distribution criteria (Kolmogorov–Smirnov test *p* > 0.05) complementary parametric tests were conducted. Therefore, an independent samples *t*-test was performed to further investigate the association between viewing time of the eyes region and the Genotype (L/L, S/−). This analysis revealed a significant effect of the 5-HTTLPR Genotype group on viewing time for the eye region [*t*(47) = 27.15, *p* = 0.008], providing evidence that Short allele carriers spent relatively less time viewing the eye region compared to participants homozygous for the Long allele. This evidence provides support for the statistical interaction observed in the initial ANOVA (see Figure 4; Table S4 in Supplementary Material).

Because a Kolmogorov–Smirnov test revealed that the data for the mouth region data were not normally distributed (*p* > 0.05), this analysis was followed up with complementary non-parametric tests using a Mann–Whitney *U* in order to further investigate the way in which 5-HTTLPR genotype groups (i.e., L/L versus S/−) differ in the time spent looking the mouth region.

This analysis revealed a significant effect of the 5-HTTLPR Genotype group on the viewing time for the mouth region (*U* = 139.0, *p* = 0.005), indicating that Short allele carriers spent relatively more time viewing the mouth region compared to participants homozygous for the Long allele. Moreover, a significant effect of genotype on looking the eye region was evident, where short allele carriers spent significant less dwell time fixating the eye region (*U* = 168.0, *p* = 0.029), when compared to carriers of two copies of the long allele. This evidence provides support for the statistical interaction (see Figure 4; Table S4 in Supplementary Material) observed in the initial ANOVA.

As a complementary analysis, we followed up the original effects of 5-HTTLPR genotype on the viewing time of the eyes versus mouth region to examine within genotype differences for looking at the eye versus mouth regions for each genotype. Therefore, we conducted additional Wilcoxon matched pairs tests which show that 5-HTTLPR S/− carriers spent significantly more time looking the mouth [*t*(31) = 16.15, *p* < 0.001] versus the eyes region [*Z* = −4.31, *p* < 0.001]. Conversely, 5-HTTLPR L/L carriers spent significantly more time looking at the eyes versus the mouth region [*Z* = −3.62, *p* < 0.001].

Repetition of the original ANOVA with additional RoIs as repeated factor (eyes, mouth, forehead, nose, cheeks, chin) confirmed the main effect of region [*F*(5,225) = 127.48, $\eta_{\text{p}}^{2}=0.73$, *p* < 0.001] and a significant two-way interaction effect between Region and 5-HTTLPR genotype [*F*(5, 225) = 3.79, $\eta_{\text{p}}^{2}=0.07$, *p* = 0.017]. To further examine the interaction effects observed in the ANOVA, and given that the data for the nose region met normal distribution criteria (Kolmogorov–Smirnov test *p* > 0.05), complementary parametric tests were conducted. Therefore, an independent samples *t*-test was performed to further investigate the association between viewing time of the nose region and the Genotype (L/L, S/−). This analysis did not revealed a significant effect of the 5-HTTLPR Genotype group on viewing time for the eye region [*t*(47) = –0.51, *p* = 0.610]. Similarly, Mann–Whitney *U*-tests for the rest of the RoIs (Kolmogorov-Smirnov test *p* > 0.05) did not show any effect of the 5-HTTLPR genotype for the viewing time spend looking the forehead (*U* = 237.5, *p* = 0.468), cheeks (*U* = 249.5, *p* = 0.636), and chin RoI (*U* = 218.5, *p* = 0.249).

## Discussion

The present study was designed to examine relationships between normal variations in genetic SNPs involved in both neural plasticity (BDNF Val^66^Met) and serotonin availability (5-HTTLPR) and visual scanning of faces in typically developing young children. It is well-established that carriers of the BDNF Met allele exhibit increased reactivity in response to emotional stimuli (Montag et al., 2008; Schofield et al., 2009; Lau et al., 2010). In a separate line of research, 5-HTTLPR Short allele carriers have been reported to exhibit increased susceptibility for psychological maladjustment and affective disorders, such as anxiety (Munafo et al., 2008; Carver et al., 2009). In the present study, young children carrying the BDNF Met allele and children carrying the 5-HTTLPR Short allele exhibited potentially reactive patterns of visual scanning of faces. Specifically, it was shown that children carrying the low activity BDNF Met allele exhibited a robust vigilance–avoidance pattern of visual scanning when processing angry but not happy faces, when compared to Val allele homozygotes. Moreover, the carriers of the low activity 5-HTTLPR Short allele spent significantly less time looking at the eye region of the face relative to the whole face, and also spent more time looking at the mouth region, compared with participants homozygous for the high serotonin activity Long allele. Although participants were not followed into adulthood, both of these findings may be relevant to later psychological difficulties and disorders.

There is increasing evidence from studies of children to suggest heightened neurophysiological sensitivity of Met allele carriers in response to negative environmental stressors (Montag et al., 2008; Schofield et al., 2009; Lau et al., 2010; Scharinger et al., 2010; Gerritsen et al., 2011). Consistent with this notion, and in line with the current study’s hypothesis, Met allele carriers spent more time fixating angry versus neutral facial expressions during early stages of processing (501–1000 ms), which decreased during later stages of processing (1501–2000 ms; 2001–2500 ms). On the other hand, participants homozygous for the high activity Val allele did not show similar patterns of avoidance. Instead, they spent significantly more time looking at the angry faces after 1500 ms, suggesting the existence of a resilience mechanism expressed through the vigilant exploration of the negative facial expression. This is consistent with previous evidence suggesting the existence of a protective mechanism against forms of psychopathology in carriers of two copies of the high neuroplasticity Val allele [e.g., Zhang et al. (2014)]. To this end, the current study provides support for the hypothesis that normal variations that modulate low neuroplasticity are associated with visual scanning pathways of emotional stimuli, perhaps due to critical influences of the BDNF Val^66^Met on the connectivity between the amygdala and the PFC (Carlson et al., 2013). However, the analyses did not reveal a similar effect of 5-HTTLPR genotype on the processing of emotional faces. While this finding may be considered inconsistent with some previous neurophysiological and behavioral studies of children and adults that have suggested 5-HTTLPR effects related to responses to emotional faces (Homberg and Lesch, 2010; Thomason et al., 2010), it is possible that developmental effects of the sample, or differences in the material used, may have contributed to these inconsistencies.

In addition to the effects of the BDNF Val^66^Met genotype in predicting preferential looking, a separate analysis suggested a role of the serotonin transporter 5-HTTLPR polymorphism in associating with gaze direction toward the eye and the mouth regions of faces posed in neutral expressions in the current study. Consistent with a plethora of studies suggesting the existence of neurobiological susceptibility for negative affectivity, such as stress reactivity, in carriers of the Short 5-HTTLPR allele [e.g., Caspi et al. (2003), Thomason et al. (2010), Mercer et al. (2012), and Disner et al. (2013)], the pattern of results of the present study showed for the first time that, early in life, the presence of the Short 5-HTTLPR may be related to face scanning behavior that has previously been associated with pervasive anxiety and/or shyness [e.g., Horley et al. (2004)]. More specifically, carriers of the low activity Short allele spent significantly less time looking at the eye region relative to the rest of the face, compared to the participants homozygous for the high serotonin activity Long allele. These individuals also spent significantly more time looking at the mouth region. Interestingly, atypicalities in allocating attention to facial features have been widely evident in various atypically developing populations, including Fragile X Syndrome, which is a disorder of both social and intellectual functioning that has been shown to be related to pervasive social anxiety and shyness (Farzin et al., 2009; Crawford et al., 2014).

One possible explanation for the observed pattern of looking behaviors is that 5-HTTLPR Short allele carriers switched their attention away from the eye region of neutral faces, engaging in a type of vigilance pattern of scanning the mouth region as a compensatory mechanism to down-regulate heightened reactivity when processing the eye region. Attention to the eyes is considered necessary for effective emotion recognition, with healthy individuals being reported to first fixate on the eyes and subsequently spend relatively more time looking at the eye region compared to the mouth region of the face (Itier and Batty, 2009). This possibility that 5-HTTLPR Short allele carriers, known to experience higher susceptibility for poor reactivity to distressing negative emotional cues, exhibit such a pattern may help to link with the literature that suggests that reduced looking to the eye region is evident in individuals with social anxiety (Horley et al., 2004; Crawford et al., 2014). The current results further suggest that the neurobiology of 5-HTTLPR Short allele carriers may contribute to this pattern of early eye gaze and, in conjunction with other factors such as negative life events, may relate to later affect-related difficulties or psychopathology.

An additional aim of the present study was to examine the relationship between processing of emotional faces and facial features with early affective symptomatology, as indexed by parent questionnaires. Contrary to the study’s hypotheses, the present results did not uncover associations between parent reports of early affective symptomatology and overall fixation duration toward emotional faces. One potential explanation for this finding may be related to the study’s sample age, which consisted of young and unaffected children compared to previous observations with older children [see Battaglia et al. (2004)] or adolescents (Gamble and Rapee, 2009). Therefore, it is plausible that as individuals are exposed to a broad range and more variable rates of stress-induced situations (e.g., school transitions), they may be more behaviorally reactive in response to environmental stressors, for example. Another possibility in this respect may be related to differences in the stimulus materials or the experimental design used in the present study. For instance, previous studies have used various negative emotional faces (Gamble and Rapee, 2009), as opposed to only angry negative emotional faces, or longer periods of angry-neutral face pair presentations (Gamble and Rapee, 2010). As Bons et al. (2013) indicate, both of these variables may be critical in shaping the patterns of findings in studies examining individual differences and may contribute to the discrepancy among studies in typical and atypical development (Bons et al., 2013).

The allocation of attention is a critical ability, which has been suggested to reflect the development and functioning of fronto-cortical circuits that ultimately reach development during the late adolescent years (Hare et al., 2005; Pessoa, 2010). Consistent with this notion, it is now widely accepted that children undergo critical periods in the formation of neural circuits that are involved in effective emotion regulation. It is critical to identify the genetic and neurobiological mechanisms contributing to behaviors that may be indicative of later psychological maladjustment early in life. In line with the findings of the current study, cognitive models of child anxiety suggest that threat avoidance may maintain anxiety in children, since children are not developing critical evaluation abilities for the formation of effective emotion regulation (Rapee, 2002; Hudson and Rapee, 2004). The present findings fill an existing gap in the literature, contributing to our understanding of the potential effect of variations in BDNF Val^66^Met and 5-HTTLPR genotypes as moderators of preferential looking toward facial expressions of threat/anger and facial features, which may suggest the existence of behavioral patterns that may link with heightened sensitivity in response to environmental stressors and the presence of psychological problems and behaviors later in life.

In summary, the current study provides evidence to suggest that carriers of the Met BDNF allele exhibited a vigilance–avoidance pattern on the dwell time looking at angry versus neutral facial expressions, compared with a high neuroplasticity homozygous Val genotype group, early in childhood. Moreover, Short allele carriers of the 5-HTTLPR polymorphism spent significantly less overall time looking at the eyes, compared with Long allele homozygotes. Taken together, these results suggest that normal variation in genetic single-nucleotide polymorphisms may contribute to the establishment of particular patterns of visual scanning toward faces and face features early in life. Overall, the outcomes of the study are consistent with existing evidence from the adult, adolescent, and child psychopathology research literatures suggesting a contribution of both BDNF Val^66^Met and 5-HTTLPR genotypes to variations in affective and emotional regulation that may be relevant to later psychological difficulties and disorders. The current findings further offer insights into particular relationships between genetic, neural, and cognitive/behavioral functions that may be related to one another in this context.

## Conflict of Interest Statement

The authors declare that the research was conducted in the absence of any commercial or financial relationships that could be construed as a potential conflict of interest.

## Supplementary Material

The Supplementary Material for this article can be found online at http://journal.frontiersin.org/article/10.3389/fnbeh.2015.00175

## Footnotes

^1^The MacBrain Face stimuli used here are as follows: *Angry Faces:* 01F, 05F, 06F, 20M, 22M; *Happy Faces:* 08F, 11F, 12F, 24M, 26M; *Neutral Faces:* 02F, 03F, 07F, 09F, 10F,13F, 14F, 15F, 16F, 17F, 18F, 19F, 21M, 23M, 25M, 28M, 29M, 30M, 31M, 32M, 33M, 34M, 35M, 36M, 37M, 38M.
