# Supplementary material for: BDNF Val66Met and 5-HTTLPR Genotype are Each Associated with Visual Scanning Patterns of Faces in Young Children
Source: Front Behav Neurosci. 2015 Jul 13;9:175. doi: 10.3389/fnbeh.2015.00175 (PMC4500100; doi:10.3389/fnbeh.2015.00175)

Supplementary information

Table S1. Participants' demographic characteristics by 5-HTTLPR genotype

		**5-HTTLPR Genotype**			**ANOVA**		
		**S/S**	**S/L**	**L/L**	***F***	**df**	***P***
**N**		10	22	17			
**Gender**	% Male*(N)*	8.2 *(4)*	20.4 *(10)*	20.4 *(10)*	*.997*	1	..*323*
	% Female *(N)*	12.2 *(6)*	24.5 *(12)*	14.2 *(7)*			

Table S2. Participants' demographic characteristics by BDNF $Val^{66}Met$ genotype

		**BDNF $Val^{66}Met$ Genotype**			**ANOVA**		
		**M/M**	**M/V**	**V/V**	***F***	**df**	***P***
**N**		3	18	28			
**Gender**	% Male*(N)*	4.1 *(2)*	16.3 *(8)*	28.5 *(14)*	*.013*	1	*.911*
	% Female *(N)*	2.1 *(1)*	20.4 *(10)*	28.5 *(14)*			

Table S3. Relative dwell time in ms and standard deviations (in brackets) viewing angry and happy faces in different genotype groups, showing an aggression-specific vigilance-avoidance patterns of attention allocation in the Met/- genotype group.

Time Interval	**BDNF**		**5-HTTLPR**	
	**M/- (N=21)**	**V/V (N=28)**	**S/- (N=32)**	**L/L (N=17)**
Facial expressions of Anger				
T1	-8 (49)	-7 (49)	-3 (45)	-16 (55)
T2	220 (138)	134 (168)	175 (156)	164 (173)
T3	292 (330.64)	438 (232.68)	399 (252)	332 (343)
T4	258 (312)	465 (237)	410 (259)	314 (336)
T5	46 (242)	191 (236)	147 (236)	94 (270)
Facial expressions of Happiness				
T1	45 (181)	18 (63)	14 (51)	60 (204)
T2	162 (291)	174 (175)	154 (193)	196 (290)
T3	181 (338)	199 (218)	172 (204)	228 (376)
T4	107 (306)	135 (251)	116 (243)	136 (331)
T5	44 (231)	23 (181)	31 (136)	33 (294)

Table S4. Means and standard deviations (in brackets) of the proportion of time each genotype group spent looking at the mouth region relative to the rest of the face during baseline trials. The S/- genotype group is spending significantly less time looking the eyes region, whereas spend more time fixating the mouth region of neutral faces.

RoI	**BDNF**		**5-HTTLPR**	
	**M/- (N=21)**	**V/V (N=28)**	**S/- (N=32)**	**L/L (N=17)**
**Eyes Region**	0.25 (0.09)	0.28 (0.09)	0.24 (0.08)	0.32 (0.10)
**Mouth Region**	0.06 (0.08)	0.04 (0.04)	0.06 (0.07)	0.02 (0.02)

Figure S1. Example of RoI selection in a neutral face stimuli

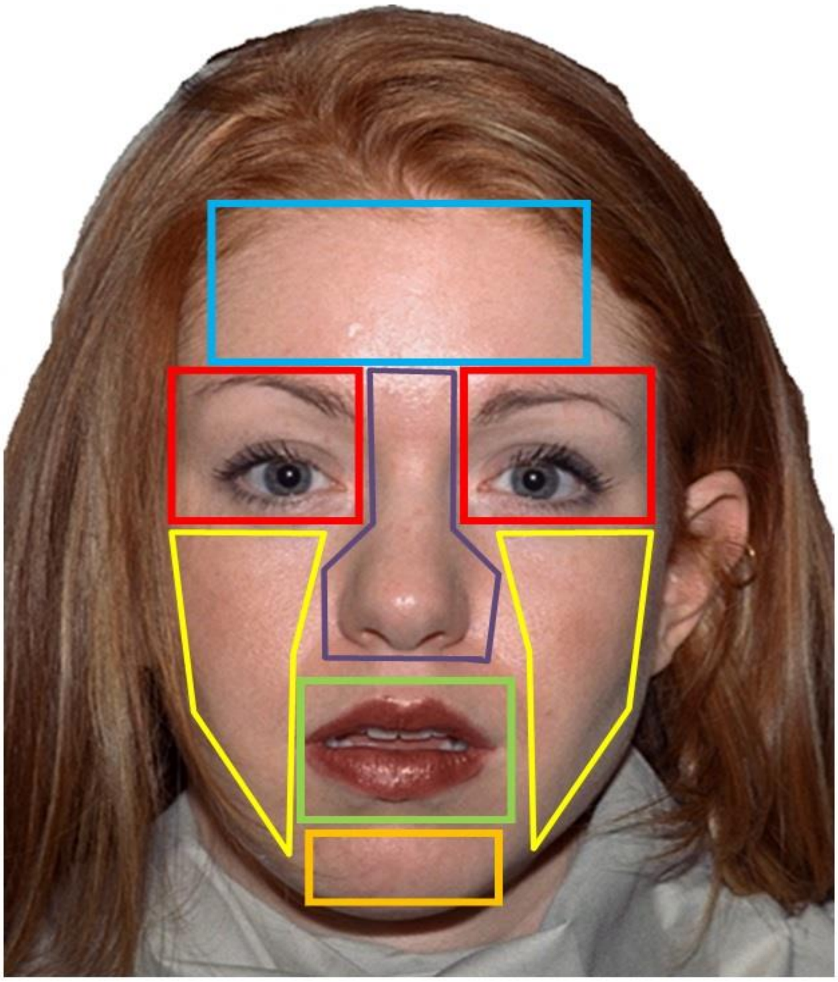

Supplement: Supplementary file 1 [file Data_Sheet_1.PDF]
